# Dexamethasone improves thymoma-associated myasthenia gravis via the AKT-mTOR pathway

**DOI:** 10.1007/s00210-023-02641-z

**Published:** 2023-07-27

**Authors:** Yuxin Liu, Si Chen, Yan Wang, Zeyang Zhang, Hui Zhang, Ziyi Wang, Ziyou Tao, Jianyao Wang, Peng Zhang

**Affiliations:** 1 Department of Cardiovascular Thoracic Surgery, Tianjin Medical University General Hospital, Tianjin, China; 2 School of Clinical Medicine, Tsinghua University, Beijing, China; 3 Department of Thoracic Surgery, The First Affiliated Hospital of Zhengzhou University, No.1 Jianshe East Road, Zhengzhou, China

**Keywords:** Thymoma, Myasthenia gravis, Dexamethasone, Network pharmacology, Molecular docking

## Abstract

Clinically, thymoma patients are often complicated with myasthenia gravis (MG). Dexamethasone, a glucocorticoid with anti-inflammatory effects, could be used as an immunosuppressant for thymoma-associated MG, but the mechanism of action remains to be explored. In this study, Gene Ontology (GO) and Kyoto Encyclopedia of Genes and Genomes (KEGG) analysis, weighted gene co-expression network analysis (WGCNA) of potential targets was performed by screening the intersection targets of dexamethasone and thymoma-associated MG from the database. Furthermore, the key targets and core active components were identified by topological analysis of the protein–protein interaction (PPI) network. Molecular docking technology was applied to screen the complexes with stable binding of dexamethasone and core targets. Patients with thymoma were divided into two groups according to whether they received dexamethasone before operation, and immunohistochemistry and western blot were used to verify the selected target of dexamethasone in treating thymoma-associated MG. The results showed that the action pathway of dexamethasone on the disease was closely enriched to phosphatidylinositol 3-kinase (PI3K)-protein kinase B (PKB/AKT), mammalian target of rapamycin (mTOR) signaling pathways. The expressions of AKT1 and its downstream molecule mTOR in the thymoma microenvironment of thymoma-associated MG patients who did not receive dexamethasone before operation were higher than those in the group receiving dexamethasone before operation. This study demonstrates that dexamethasone can promote apoptosis through the AKT-mTOR pathway for the treatment of thymoma-associated MG, as validated by network pharmacology predictions and clinical specimen experiments, and can be verified by large-scale clinical trials in the future. This study also provides theoretical support and new research perspectives for this disease.

**Supplementary Information:**

The online version contains supplementary material available at 10.1007/s00210-023-02641-z.

## Introduction

Some autoimmune diseases are common in 30–40% of patients with thymoma (Zekeridou et al. 2016), and younger patients with thymoma are more likely to have autoimmune diseases including myasthenia gravis (MG) (Radovich et al. 2018). The complexity of the components of the thymus as a central immune organ determines the close relationship between thymoma and autoimmunity. MG persists after thymoma resection in a few patients with thymoma-related MG. Immunotherapy and new targeted therapies have shown promising efficacy in thymoma and thymic carcinoma (Conforti et al. 2020).

MG is an autoimmune disease of the neuromuscular junction characterized by skeletal muscle weakness (Lefeuvre et al. 2020). There is a clear association between MG and thymoma (Ruan et al. 2021). However, the causal relationship and mechanism of action between thymoma and MG remain unclear, and whether MG is a risk factor for thymoma remains controversial. Early removal of thymoma is essential for the treatment of thymoma-related MG (Wolfe et al. 2016). Patients with thymus-associated MG often have more severe symptoms and require more immunosuppressive therapy (Lefeuvre et al. 2020). Dexamethasone is a glucocorticoid with good anti-inflammatory and immunosuppressive effects. The main role of glucocorticoids in thymoma-associated MG is to prevent B cells from producing new plasma cells and impairing the activation and proliferation of T cells (Gomez et al. 2014). Studies have shown that ligand-bound glucocorticoid receptors inhibit T-cell receptor (TCR) activation by inhibiting the synthesis of interleukin (IL)-2 via interfering with transcription factors that regulate cytokine gene expression (Harr et al. 2009). Evidence shows that dexamethasone could downregulate IL-21 and thus inhibit IL-21/ transcription (STAT)3/B-lymphocyte-induced maturation protein 1 (Blimp1) to prevent B cells from generating new plasma cells, which is an important way to treat MG (Xu et al. 2021). A review of the literature has shown that patients with thymoma treated with glucocorticoids (particularly dexamethasone) have experienced tumor resolution and symptomatic relief (Barratt et al. 2007). Studies have shown that dexamethasone reduces tumor volume, vascular invasion, and levels of the proliferation markers Ki67 and c-Myc, as well as the anti-apoptotic marker Bcl2 (Xu et al. 2020),dexamethasone inhibits TRAIL-mediated apoptosis via GSK-3β-mediated DR5 downregulation and c-FLIP(L) upregulation in cancer cells (Jeon et al. 2020). Tacrolimus, a calcineurin inhibitor, is beneficial in the treatment of some MG patients by affecting muscle contraction (regulating intracellular calcium release channels and ryanodine receptors that increase muscle strength), glucocorticoid receptors (increasing intracellular concentrations of steroids and blocking steroid export mechanisms), and increasing T-cell apoptosis (Ponseti et al. 2008), with long-term use significantly reducing the need for oral steroids in MG patients (Alhaidar et al. 2022). Therefore, it is urgent to study whether there is a therapeutic mechanism of dexamethasone for thymoma-related MG.

In this study, the Pubchem database was used to obtain the target of dexamethasone, and the Gene Cards database was used to obtain the disease target of thymoma-related MG. In addition, the topological analysis of protein–protein interaction (PPI) network was used to screen out the duplicate targets of the two. Gene Ontology (GO) and Kyoto Encyclopedia of Genes and Genomes (KEGG) enrichment analysis was performed to further explore the molecular mechanism of dexamethasone in the treatment of thymoma-associated MG. We predicted dexamethasone targeted important signaling pathways in thymoma-associated MG and the biological processes involved. Finally, we performed molecular docking verification on the screened core target genes and validated in the patient tumor microenvironment (TME). Based on the above studies, our purpose is to provide a reference for the clinical treatment of thymoma-associated MG and to further reveal the potential mechanism of dexamethasone in the treatment of thymoma-associated MG.

## Materials and methods

### Drug structure search and target prediction

**Fig. 1:**
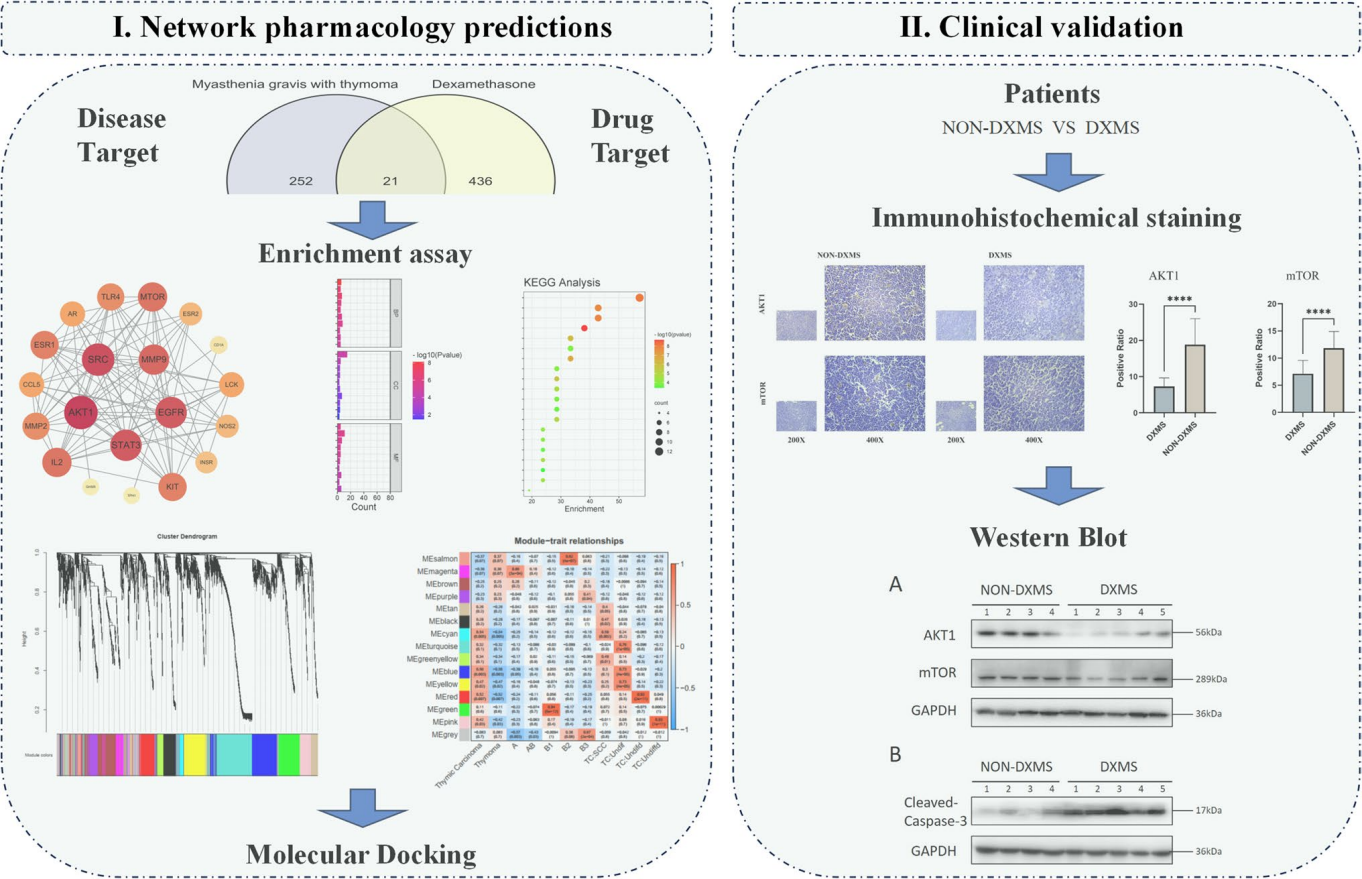
Workflow of the study

The dexamethasone was searched from Pubchem database and inputted to PharmMapper (http://www.lilab-ecust.cn/pharmmapper/) and Super-PRED (https://prediction.charite.de/index.php?site=chemdoodle_search_target) platform to predict all the corresponding targets of it (Yuan et al. 2022). The workflow is shown in Fig. 1.

### Disease target screening

The human gene database (GeneCards, https://genealacart.genecards.org/) (Safran et al. 2010) was used to search for this disease targets with “Myasthenia gravis with thymoma” as the search term. The disease targets retrieved from the database were combined and deduplicated to serve as the final target source for thymoma-associated MG.

### PPI network analysis

The screened dexamethasone targets and thymoma-associated myasthenia gravis targets were screened, and duplicated targets were screened. Inputting the intersection targets into the STRING database (https://cn.string-db.org/) (Szklarczyk et al. 2017), we selected Multiple proteins, limited the species to Homo Sapiens, constructed the PPI functional protein action network, and drew the network map.

In order to screen core proteins, the generated TSV files were imported into Cytoscape 3.7.2 for topology analysis (Shannon et al. 2003), and the top 5 degree values were selected as key targets.

### GO-KEGG enrichment analysis

The common targets of drugs and diseases were imported into DAVID Bioinformatics Resources 6.8 (https://david.ncifcrf.gov/home.jsp), and all targets were subjected to GO biological process (BP), cell component (CC) enrichment, and molecular function (MF) enrichment analysis. The enrichment conditions were *P* value cutoff = 0.05 and *Q* value cutoff = 0.05. The top 10 enriched items by *P* value were selected, and WeChat(http://www.bioinformatics.com.com.cn/) was used for visualized analysis of the results through the R package according to the *P* value, *Q* value, and the number of genes enriched on each item. (Heatmap was plotted by https://www.bioinformatics.com.cn, a free online platform for data analysis and visualization.)

Then carrying out KEGG pathway enrichment analysis on the common targets of drugs and diseases, the common targets of drugs and diseases were imported into DAVID Bioinformatics Resources 6.8 (https://david.ncifcrf.gov/home.jsp) for KEGG pathway enrichment of the targets and set analysis; items with corrected *P* value < 0.05 were screened. The top 20 enriched items by *P* value were selected, and WeChat (http://www.bioinformatics.com.com.cn/) was performed for visualization of results using the R package according to the *P* value, *Q* value, and the number of genes enriched on each item. (Heatmap was plotted by https://www.bioinformatics.com.cn, a free online platform for data analysis and visualization.)

### Weighted gene co-expression network analysis (WGCNA)

We picked another dataset GSE57892 (Petrini et al. 2014) for Weighted Gene Co-Expression Network Analysis (WGCNA) analysis (Langfelder and Horvath 2008). Genes with a variance of 0 were filtered, and then a total of 3211 genes with the top 15% of the variance were selected for subsequent analysis. The soft threshold power of *β* was 10. After converting the adjacency matrix to a topological overlap matrix (TOM), a hierarchical clustering dendrogram of genes was used to identify co-expression modules. Module eigengenes (ME) and correlations between ME and clinical information were calculated in order to mine the gene expression patterns of multiple samples and verify the hub genes related to thymoma.

### Molecular docking

Using the PubChem database to determine the compound name, molecular weight, and 2D structure of the active ingredient, then the 2D structure corresponding to the active ingredient was downloaded from the RCSB PDB database (http://www.rcsb.org/). Then AutoDock Vina software (http://vina.scripps.edu/) was used to prepare ligands and proteins required for molecular docking. For the target protein, its crystal structure need to be pretreated, including removal of hydrogenation, modification of amino acids, optimization of energy, and adjusting the force field parameters, after which the low-energy conformation of the ligand structure was satisfied. Finally, the target structure was molecularly docked with the active ingredient structure, and the vina inside the pyrx software was used for docking. The affinity value (kcal/mol) represented the binding ability of the two. The lower the binding ability, the more stable the binding of the ligand to the receptor. Pymol was used for visualization analysis, and the 2d image was visualized analysis with Discovery Studio 2020 Client.

### Patients

All the specimens were acquired following the Declaration of Helsinki and adherence to guidelines from the local ethical committee (IRB2019-KY-179). In this study, we recruited the patients who had confirmed diagnoses of thymoma-associated MG and had undergone surgical resection of thymoma in the Department of Cardiothoracic Surgery, General Hospital of Tianjin Medical University. The participants were divided into two groups according to whether dexamethasone was used before surgery. Tissue specimens from the enrolled patients were collected, soaked in formalin, embedded in paraffin, and subjected to immunohistochemical staining.

### Immunohistochemical staining

The paraffin sections of thymoma tissues of the samples were dewaxed with xylene and hydrated with different concentrations of ethanol, followed by antigen retrieval; then endogenous peroxidase blocking agent was incubated. Primary antibody mTOR (Cell Signaling Technology, 2983S) or AKT1 (Abcam, ab238477) was added dropwise and incubated overnight. Tissue sections were incubated with secondary antibody (goat anti-mouse/rabbit IgG polymer, ZSGB-BIO, PV6000). 3,3′-Diaminobenzidine (DAB) chromogenic solution (ZSGB-BIO, ZLI-9018) was added to each slice to develop color. After rinsing with water, the tissue sections were counterstained with hematoxylin staining solution. Afterwards, the sections were dehydrated with alcohol, cleared with xylene, and fixed with neutral glue. The staining results were observed under a microscope.

### Western blot

The thymic tumor tissues of the enrolled patients were subjected to Radio Immunoprecipitation Assay (RIPA) lysis buffer (C1053, BeiJing Applygen Technologies Inc.) to extract total protein. Protein concentration was quantified by bicinchoninic acid (BCA) assay kit (Beyotime Biotechnology). Then, the lysate supernatant was heated in sodium dodecyl sulfate–polyacrylamide gel electrophoresis (SDS-PAGE) sample-loading buffer (Beyotime Biotechnology). Protein extracts were separated on 8–15% sodium dodecyl sulfate (SDS)-polyacrylamide gels and transferred to the nitrocellulose membranes (Merck Millipore). The protein was then probed with a specific primary antibody followed by a horseradish peroxidase-conjugated antibody. Antibodies used were as follows: anti-AKT1 (Abcam, ab238477), anti-mTOR (Cell Signaling Technology, 2983S), anti-Cleaved-Caspase-3 (Abcam, ab2302), and anti-glyceraldehyde-3-phosphate dehydrogenase (GAPDH) (Beyotime Biotechnology, AF5009). Chemiluminescence was captured using an automatic digital gel image analysis system Tanon-4500.

### Statistical analysis

Data were checked for normality with the Kolmogorov–Smirnov or Shapiro–Wilk test; the mean difference between the two groups was tested using 2-dependent sample Student’s *t*-test if the variables were normally distributed. Data were expressed as the mean ± standard deviation (SD). Significance was considered at an alpha value of 0.05. All statistical analyses were performed by SPSS V26.0. The Prism software (GraphPad Software) version 8 was used for data analysis and graphing data.

## Results

### Screening of shared targets of dexamethasone and thymoma-associated myasthenia gravis

**Fig. 2:**
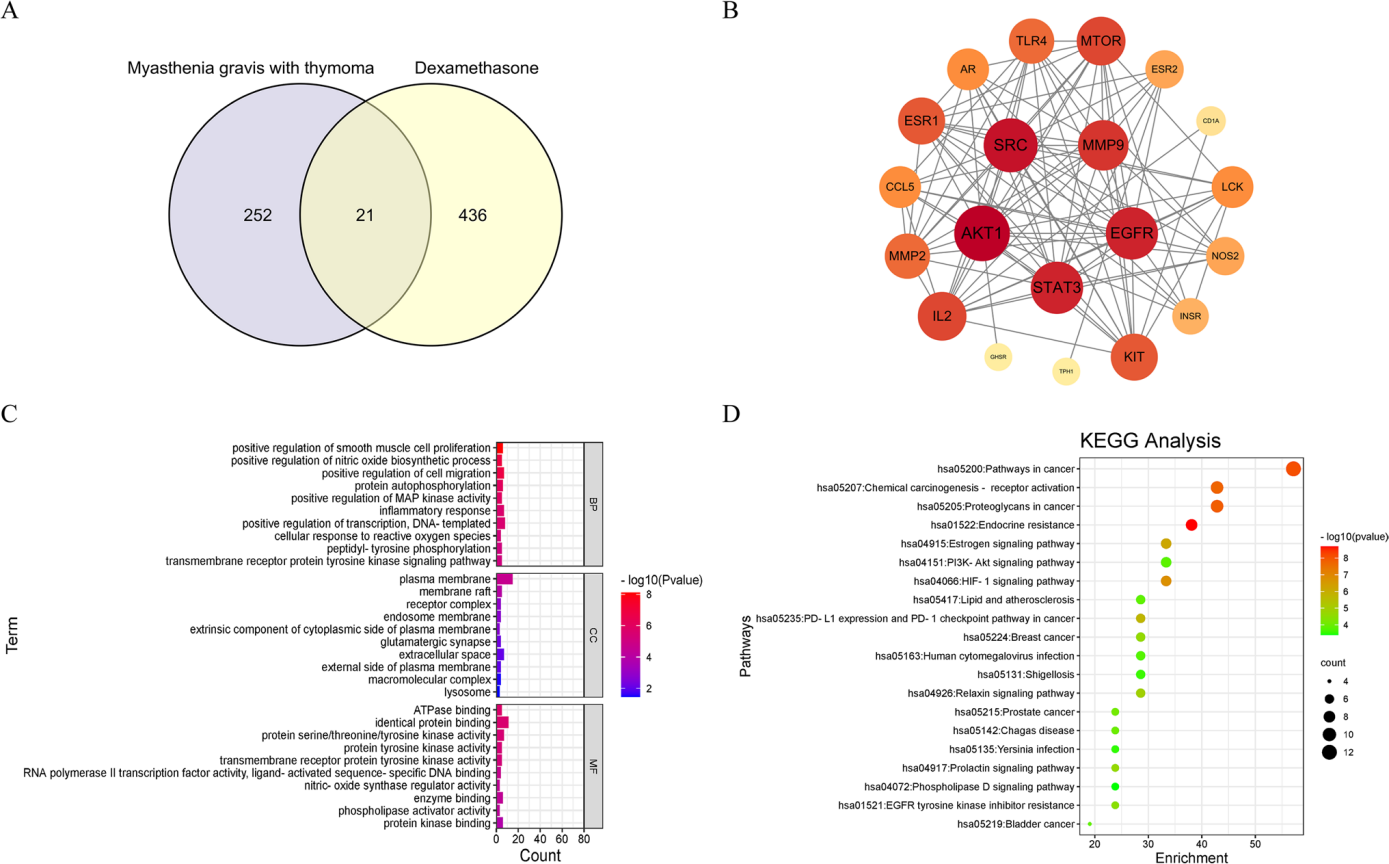
Mining of target genes. **A** Venn diagram showing the communicative targets of dexamethasone and thymoma-associated myasthenia gravis. **B** PPI protein interaction network diagram. **C** GO enrichment analysis results of disease and drug shared targets. **D** KEGG enrichment analysis results of disease and drug shared targets

We searched the compound dexamethasone by using the Pubchem database and entered it into PharmMapper (http://www.lilab-ecust.cn/pharmmapper/) and Super-PRED (https://prediction.charite.de/index.php?site=chemdoodle_search_target) platform to predict the corresponding targets, and the results showed that there were 457 corresponding targets for dexamethasone. With “Myasthenia gravis with thymoma” as the search term, relevant disease targets were searched using the Human Gene Database (GeneCards, https://genealacart.genecards.org/). The disease targets retrieved from the database were combined and deduplicated as the final source of disease targets. After deduplication, there were a total of 273 targets. The screened targets of dexamethasone and thymoma-associated myasthenia gravis were screened, and a total of 21 intersecting targets were finally obtained. (Fig. 2A).

### PPI protein interaction network analysis

All the obtained intersection targets were entered into the STRING database (https://cn.string-db.org/), with Multiple proteins selected, the species limited to Homo Sapiens, and the PPI functional protein interaction network constructed. In order to screen core proteins, the generated TSV files were imported into Cytoscape 3.7.2 for topology analysis, and the top 5 degree values were selected as key targets. (Table 1) (Fig. 2B).

**Table 1:** Key target screening results

Target	Degree
AKT1	17
SRC	16
STAT3	15
EGFR	15
MMP9	14

### GO-KEGG enrichment analysis

The shared targets of dexamethasone and thymoma-associated myasthenia gravis were imported into DAVID Bioinformatics Resources 6.8 (https://david.ncifcrf.gov/home.jsp) targets for GO enrichment analysis. The enrichment conditions were *P* value cutoff = 0.05 and *Q* value cutoff = 0.05. Our analysis found that a total of 231 GO entries were enriched. Among them, there were 18 cellular components (CC), which were mainly located in the plasma membrane, membrane rafts, receptors, endosomal membranes, exogenous components on the cytoplasmic side of the plasma membrane, glutamatergic synapses, extracellular space, and the external side of the plasma membrane, complex macromolecules, and lysosomes. There were 181 biological processes (BP), which mainly regulated and participated in the positive regulation of smooth muscle cell proliferation, positive regulation of nitric oxide biosynthesis, positive regulation of cell migration, protein autophosphorylation, positive regulation of MAP kinase activity, inflammation Response, positive regulation of transcription, DNA template, cellular response to reactive oxygen species, transmembrane receptor protein tyrosine kinase signaling pathway, peptidyl-tyrosine phosphorylation regulation, and other processes. There were 32 molecular functions (MF), which mainly were embodied in ATPase binding, the same protein binding, protein serine/threonine/tyrosine kinase activity, transmembrane receptor protein tyrosine kinase activity, RNA polymerization enzyme II transcription factor activity, ligand-activated sequence-specific DNA binding, nitric oxide synthase modulator activity, enzyme binding, phospholipase activation, and protein kinase binding regulation. Selecting the top 10 enriched items by *P* value, the results were visualized according to the *P* value, *Q* value, and the number of genes enriched on each item, as shown in Fig. 2C.

KEGG pathway enrichment analysis showed that a total of 73 signaling pathways were enriched. The top 20 enriched entries with *P* value were selected for visual analysis of the results. These pathways mainly involved endocrine resistance, cancer pathways, chemical oncogenic-receptor activation, HIF-1 signaling pathway, estrogen signaling pathway, PD-L1 expression in cancer and PD-1 checkpoint pathway, relaxin pubis signaling pathway, the prolactin signaling pathway, and PI3K-AKT and other signaling pathways, and the results are shown in Fig. 2D.

### Co-expression gene verification

**Fig. 3:**
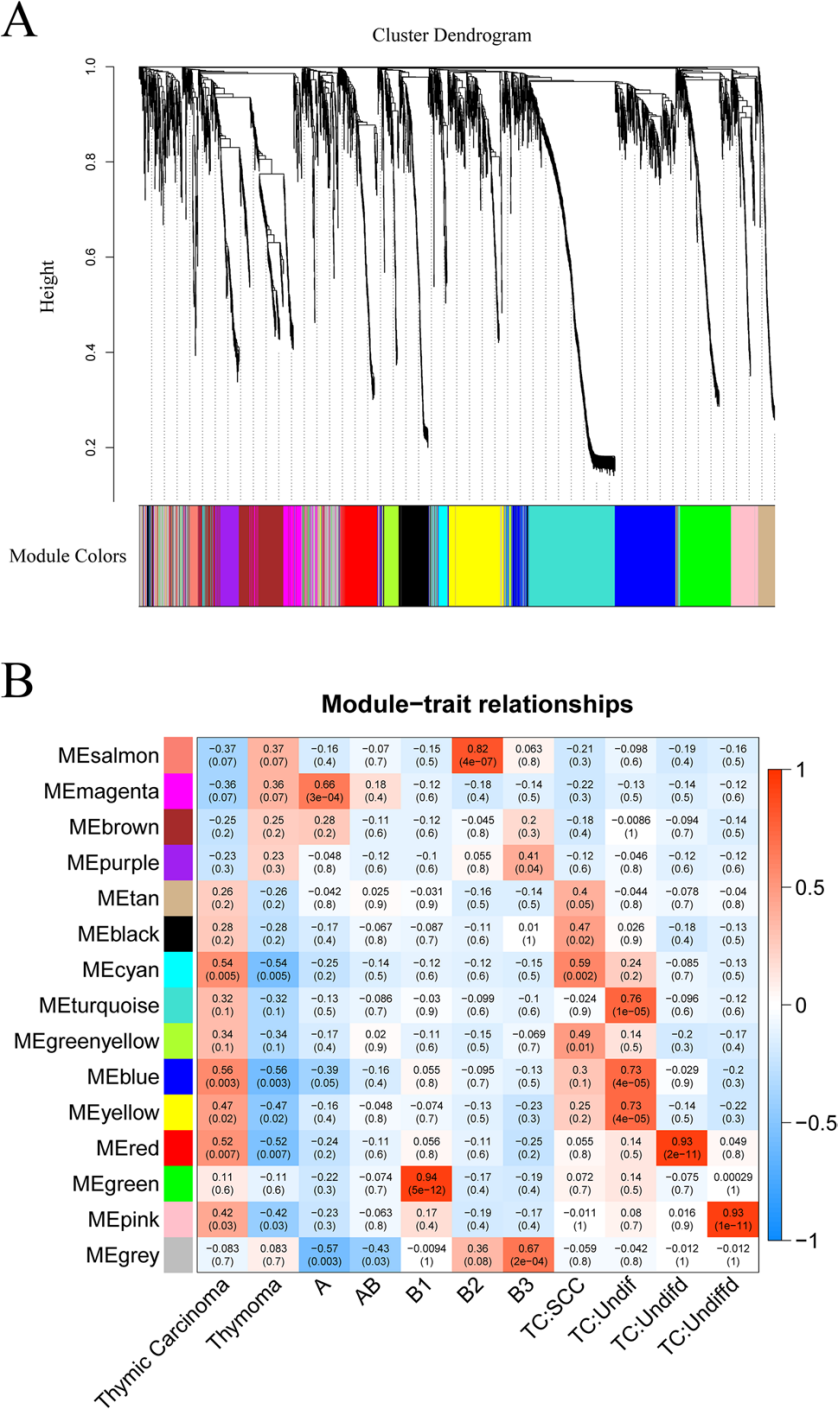
Identification of modules linked to clinical features of thymoma. **A** Cluster dendrogram of co-expressed genes in thymoma. **B** Heat map of module–trait relationships in thymoma

We selected the dataset GSE57892 from the GEO database for WGCNA analysis, drew a heat map of the module-trait relationship to evaluate the relationship between modules according to the Spearman correlation coefficient (Fig. 3A), and verified the hub gene in thymoma. According to the variance results of thymoma gene expression, the top 15% genes with larger variance were selected for co-expression analysis. A co-expression network consisting of 3211 genes was constructed. A soft threshold of 10 was chosen, and a total of 15 co-expression modules were identified. We found that AKT1 was enriched in the MEpurple module and was highly associated with type B3 thymomas (Fig. 3B).

### Molecular docking model validation

In order to clarify the binding activity between the core target protein of the disease and the active ingredient of dexamethasone, with dexamethasone used as the ligand, and the core targets AKT1, MMP9, STAT3, SRC, and EGFR used as receptors, AutoDock Vina software was used to prepare the ligands and proteins required for molecular docking, and Vina inside the pyrx software was used for docking verification. The affinity value (kcal/mol) represented the binding capacity of the two. The lower the binding capacity, the more stable the ligand-receptor binding. The binding energy predictions of the obtained five key targets and dexamethasone are shown in Table 2.

**Table 2:** Ingredient and target binding energy

Ligand/protein	AKT1	EGFR	MMP9	SRC	STAT3
Binding energy (kcal/mol)	− 9.8	− 7.8	− 8.5	− 8	− 8.3

**Fig. 4:**
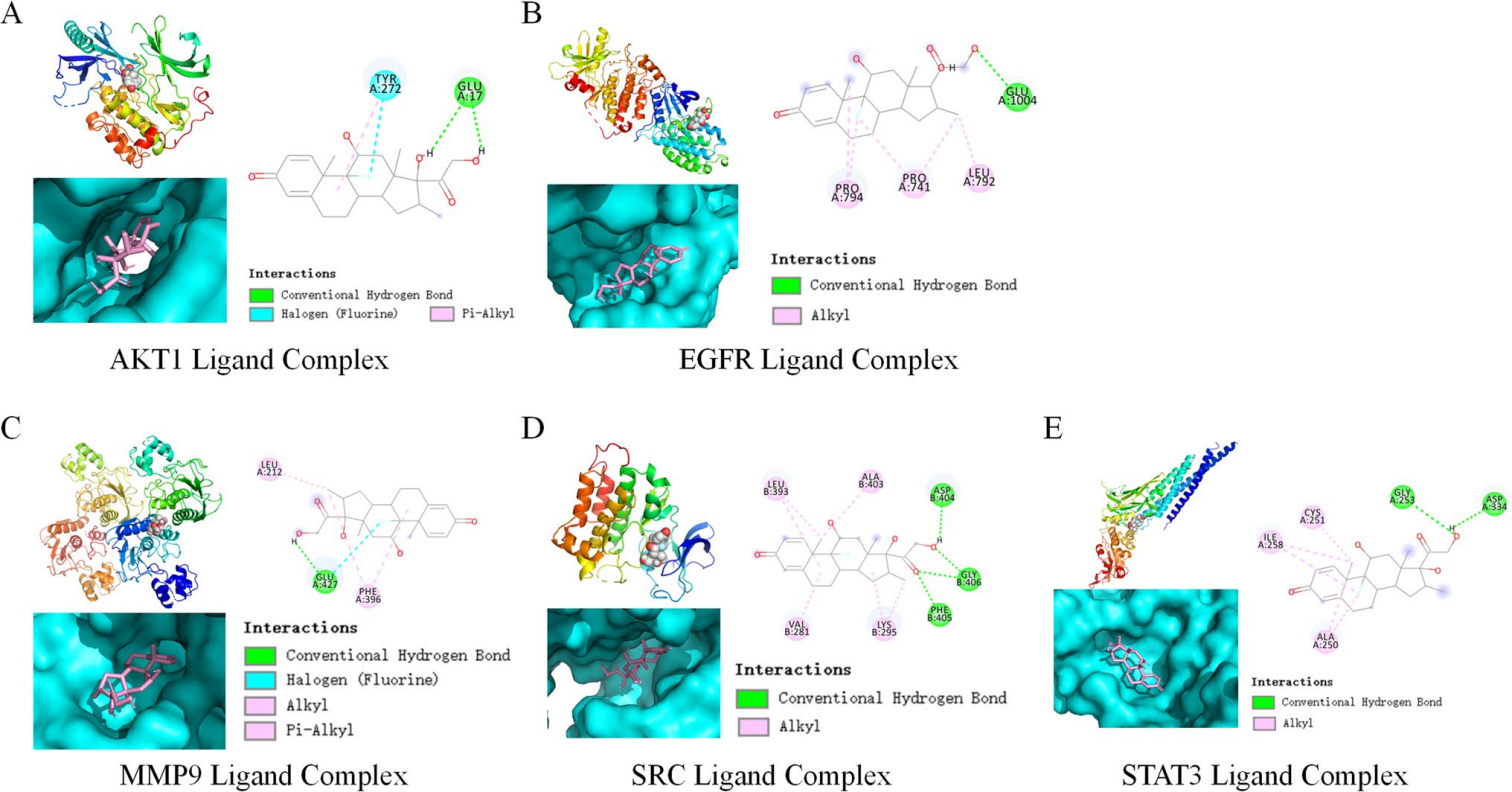
Visual analysis of molecular docking between active components of dexamethasone and core targets of thymoma-associated myasthenia gravis

The results showed that the docking energy values of AKT1, MMP9, STAT3, SRC, and EGFR were all less than − 7.0 kcal/mol, indicating that they had strong binding activity, suggesting that it was predicted that dexamethasone could have the treatment of thymoma-associated myasthenia gravis via AKT1, MMP9, STAT3, SRC, EGFR, and other targets. The docking results showed that it had good binding activity, which preliminarily verified the results of network pharmacology mining. Pymol software was conducted to visualize the docking results (Fig. 4). Based on the binding mode of molecular docking of AKT1 and ligand, AKT1 formed salt bridge interaction and Pi-alkyl hydrophobic interaction with TYR272 of the target protein of ligand and formed hydrogen bond interaction with GLU17. The docking results showed that the docking binding energy of RR1 and ligand was − 9.8 kcal/mol. Based on the binding mode of molecular docking between EGFR and ligand, EGFR formed hydrogen bond interaction with GLU1004 of the target protein of ligand and formed alkyl hydrophobic interaction with PRO741, PRO794 and LEU792. The docking results showed that the docking binding energy of EGFR and ligand was − 7.8 kcal/mol. Based on the binding mode of molecular docking MMP9 and ligand, MMP9 formed salt bridge and hydrogen bond interaction with GLU427 of the target protein of ligand, formed Pi-alkyl hydrophobic interaction with PHE396, and formed alkyl hydrophobic interaction with PHE396 and LEU212. The docking results showed that the docking binding energy of MMP9 and ligand was -8.5 kcal/mol. Based on the binding mode of molecular docking SRC and ligand, SRC formed hydrogen bond interactions with ASP404, GLY406, and PHE405 of the target proteins of ligand and formed alkyl hydrophobic interactions with LEU393, ALA403, VAL281, and LYS295. The docking results show that the docking binding energy of SRC and ligand is − 8 kcal/mol. Based on the binding mode of molecular docking between STAT3 and ligand, STAT3 formed hydrogen bond interactions with GLY253 and ASP334 of ligand target proteins and formed alkyl hydrophobic interactions with ALA250, CYS251, and ILE258. The docking results showed that the docking binding energy of STAT3 and ligand was − 8.3 kcal/mol.

### Preliminary research on the results of pathway verification

We systematically and retrospectively analyzed 12 patients who were diagnosed with thymoma-associated MG in the Department of Cardiothoracic Surgery of Tianjin Medical University General Hospital and had undergone surgical resection of thymoma. They were divided into 2 groups according to whether dexamethasone was used before surgery. The clinical information of the enrolled patients is shown in Supplementary Table 1. The thymoma tissues of the enrolled patients were selected to verify the expression of AKT1 and mTOR.

**Fig. 5:**
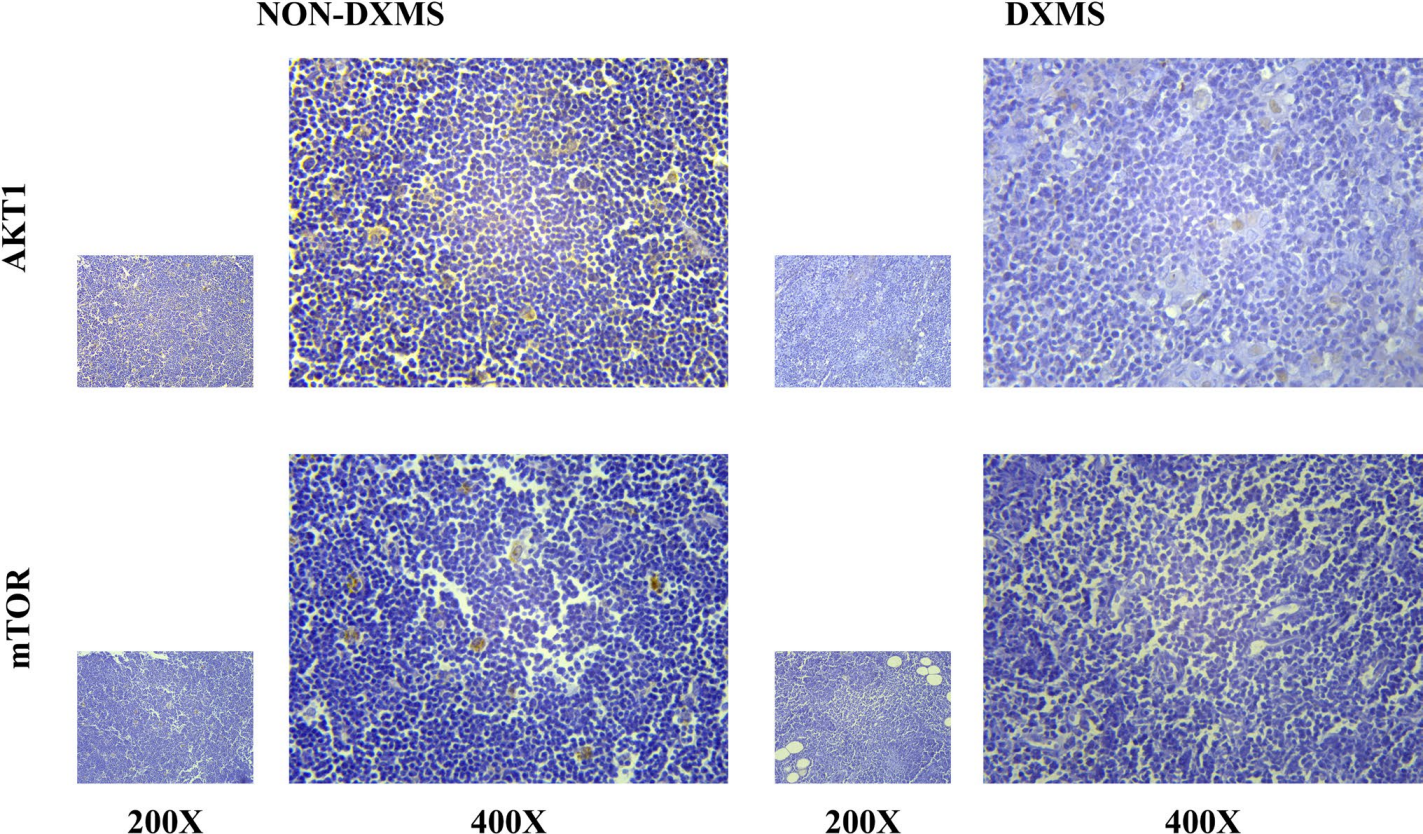
Expression of AKT and mTOR in the thymoma TME. NON-DXMS, non-dexamethasone treatment group. DXMS, dexamethasone treatment group

**Fig. 6:**
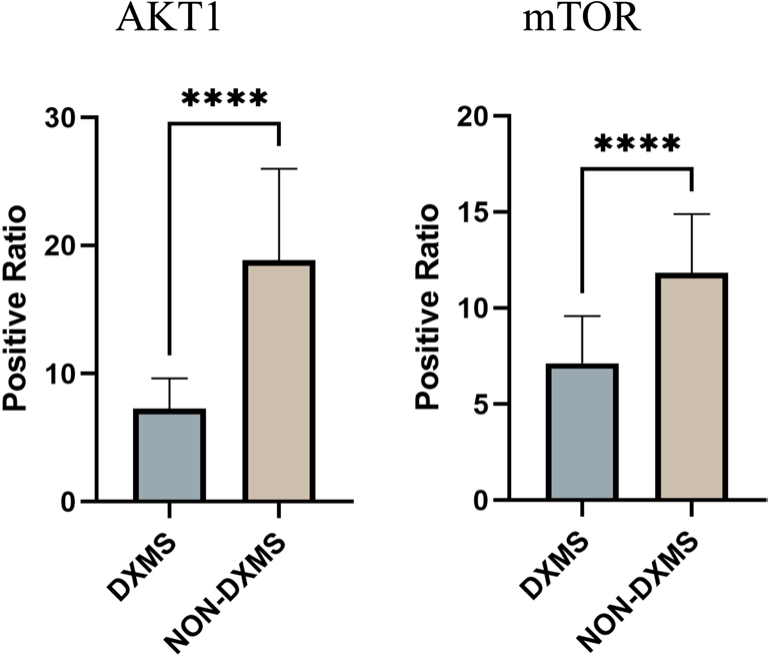
Statistical results of AKT1 and mTOR expression in thymoma tissue. Each tissue section was randomly taken with 5 fields of view, and the positive ratio was analyzed using ImageJ. Data are presented as mean ± SD (5 fields per sample, *n*_DXMS_ = 4, *n*_NON-DXMS_ = 8) of the representative data from three independent experiments (****P < 0.001)

Statistics showed that the expressions of AKT1 and mTOR in the thymoma TME of the dexamethasone-treated group were lower than those of the non-dexamethasone-treated group (Figs. 5 and 6).

**Fig. 7:**
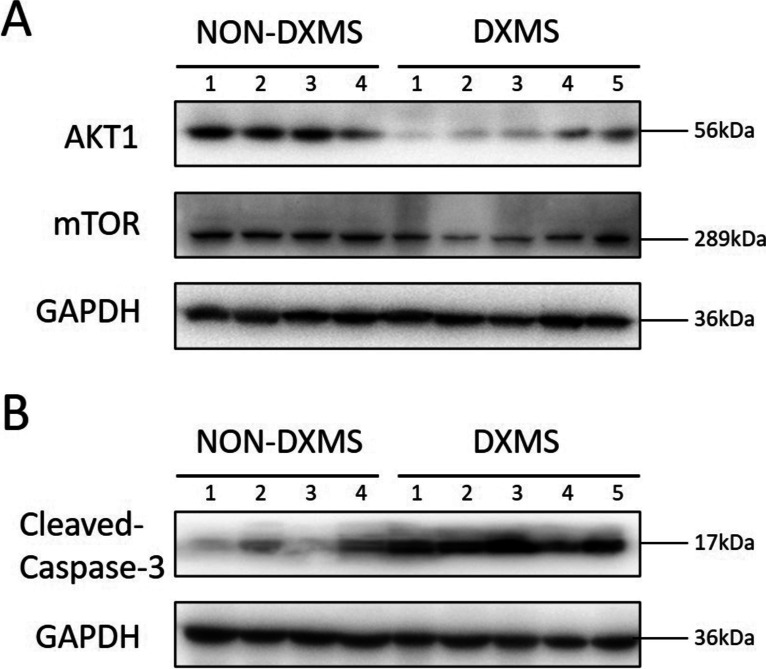
Western blot analysis results

Then we detected the level of AKT1 apoptosis pathway-related proteins in tissues by Western Blot (Fig. 7). The results showed that the level of AKT1 and mTOR protein in all 5 thymoma patients who received DXMS treatment was lower than that in the non-DXMS group (Fig. 7A), while the level of apoptosis protein, cleaved caspase-3, showed the opposite trend (Fig. 7B).

Western blot analysis was used to evaluate the protein expression level of the AKT1, mTOR, and cleaved-caspase-3 in the DXMS group and the NON-DXMS group. The western blot results of all the enrolled patients are shown in the figure.

## Discussion

Thymoma is closely related to MG, up to 10% to 15% of MG patients are combined with thymoma, and 30% to 45% of thymoma patients are combined with MG. Almost all MG patients with thymoma have anti-AChR antibodies (Álvarez-Velasco et al. 2021). At present, surgical resection is the main treatment for thymoma-associated myasthenia gravis, and hormone therapy is only used as an adjuvant treatment. During the early years, Huard and Andrea Doria et al. described that high-dose dexamethasone treatment was beneficial for the treatment of myasthenia gravis by significantly reducing B-cell activating factor (BAFF) and its mRNA in circulating blood (Huard et al. 2001), (Zen et al. 2011). In conjunction with our findings in this study, the therapeutic mechanism of dexamethasone for thymoma-associated MG was to mediate the AKT-mTOR pathway to induce apoptosis.

Based on network pharmacology, we found that AKT1 was the core target of dexamethasone in the treatment of thymoma-associated MG. AKT1 was also confirmed to have the highest binding energy with dexamethasone through molecular docking technology. Furthermore, KEGG pathway enrichment showed the PI3K-AKT pathway was also one of the hub pathways. Meanwhile, the inflammatory response and immune effects were regulated from various aspects by affecting the function of immune cells (Zhang et al. 2022), including functions affecting various B cells, such as marginal zone B cell proliferation, IgG conversion, and plasma blast differentiation (Sintes et al. 2017). It could also affect the differentiation of primitive CD4 + T cells so as to treat myasthenia gravis (Pae and Wu 2013). Thus, we continued to verify the positive effect of dexamethasone on thymoma-associated MG focusing on the AKT1 and its downstream factor mTOR. The structure of the thymus is complex, and there are many components of immune cells.

Some studies have shown that AKT1 increased in patients with myasthenia gravis (Maurer et al. 2015; Fang et al. 2022), encoded serine-threonine protein kinase, which affected many biological processes including proliferation, metabolism, and angiogenesis (Sanchez-Gurmaches et al. 2019). P13K-AKT, as the most main signaling pathways, played a vital role in B cell proliferation, differentiation and apoptosis, glucose transporter, and T cell development (Zhang et al. 2022; Jellusova and Rickert 2016). It is showed that AKT inhibitor could upregulate the number of Th1 and Th17 cells in peripheral blood of patients with myasthenia gravis, suggesting that AKT inhibitor could regulate the immune function of patients with myasthenia gravis (Feng and Qiu 2018).

Aberrant overexpression or activation of AKT is observed in many cancers, including ovarian, lung, pancreatic cancers (Song et al. 2019), and thymoma, and is associated with increased cancer cell proliferation and survival. Therefore, targeting AKT may provide an important avenue for cancer prevention and treatment.

Our study showed that in the thymoma TME, the expressions of AKT1 and mTOR in the non-dexamethasone treatment group were higher than those in the dexamethasone treatment group. Studies have shown that dexamethasone inhibited the migration and invasion of non-small-cell lung cancer by regulating the AKT signaling pathway (Zhang et al. 2020). Han et al. (Han et al. 2016) found that dexamethasone regulated ERK and AKT in human colon cancer cells to inhibit cell migration. In the inflammation-induced mechanism of acute radiation enteritis, dexamethasone can also inhibit inflammation by inhibiting AKT-related pathways (Li et al. 2022).

The mechanism of action of prednisolone in thymoma-associated MG is to prevent the formation of GC, but not the production of antibodies by thymic B cells, and to reduce the expression of chemokines (CXCL 13, CCL 21, and CCL 19) and the number of HEV (Truffault et al. 2017). Studies have shown that in thymoma-associated MG, preoperative application of prednisolone to improve symptoms of muscle weakness may result in radiological shrinkage of the thymoma, with pathological manifestations of marked loss of thymic lymphoid tissue but preservation of epithelial components (Zouvelou et al. 2022).

Some studies have shown that high doses of prednisone and prednisone exacerbate MG symptoms, and some scholars have suggested the use of alternate days of high doses (Warmolts and Engel 1972) or gradually increasing doses (Seybold and Drachman 1974) of oral or intravenous regimens (Utsugisawa et al. 2017), but both have also been associated with initial deterioration (Lotan et al. 2021). Patients with thymoma have been treated with glucocorticoids (especially dexamethasone) for tumor resolution and symptomatic relief (Barratt et al. 2007). Dexamethasone peribulbar or extraocular muscle injections are effective in treating patients with oculomotor MG and can be an alternative to systemic medication (Shi et al. 2020). Taken together, our findings suggest that dexamethasone should be given a higher status than other steroids in the pharmacological treatment of myasthenia gravis. We also speculate on the treatment of patients with thymoma-related MG and whether the choice of glucocorticoids is to replace other steroids with dexamethasone or to combine it with other steroids. Future large-scale clinical studies on dexamethasone in the treatment of thymoma-associated MG could provide new clinical perspectives.

Altered T cell development within the thymus is thought to be associated with autoimmune disease progression in thymoma patients (Scorsetti et al. 2016). Treatment of TMG mainly relies on the combined use of glucocorticoids and immunosuppressants, which mainly work by preventing B cells from producing new plasma cells and impairing the activation and proliferation of T cells (Gomez et al. 2014). Studies have shown that dexamethasone induced osteoblast apoptosis through the ROS-PI3K/AKT/GSK3β signaling pathway (Deng et al. 2019). Our results also confirmed that the expression level of AKT1 and mTOR were decreased and the expression level of the apoptosis-related protein, cleaved-caspase-3, was increased in the TME of thymoma patients using DXMS, which also suggested that DXMS promoted apoptosis of thymoma cells. Another study showed that the expressions of PI3K, p-AKT, and p-mTOR were all suppressed in primary osteoclasts under dexamethasone-induced autophagy. Activation of the PI3K/AKT/mTOR pathway using the selective PTEN inhibitor SF1670 reversed this osteoclast autophagy under dexamethasone treatment (Fu et al. 2020). This also just confirmed the previous research conclusions. Dexamethasone may inhibit thymoma-associated myasthenia gravis through the AKT-mTOR pathway. More mechanisms need to be verified by in vitro experiments.

Although we completed a pathway validation from enrichment analysis of network pharmacology to clinical specimens, it still has some limitations. First, because of the breadth of clinical use of dexamethasone, we lacked a certain patient population base, which resulted in the small number of clinical samples we included. Secondly, we have only completed validation in the clinical sample at our center, and deeper mechanistic exploration relies on the development of cellular and animal experiments. Thirdly, due to the limitation of the sample size, this study only provides a preliminary theoretical basis and has not yet explored in depth the effects of different doses of dexamethasone on patients. Therefore, in the future, the design of large-scale clinical trials should be improved for different doses and different tissue levels, so as to provide new ideas for clinical treatment protocols.

## Conclusion

**Fig. 8:**
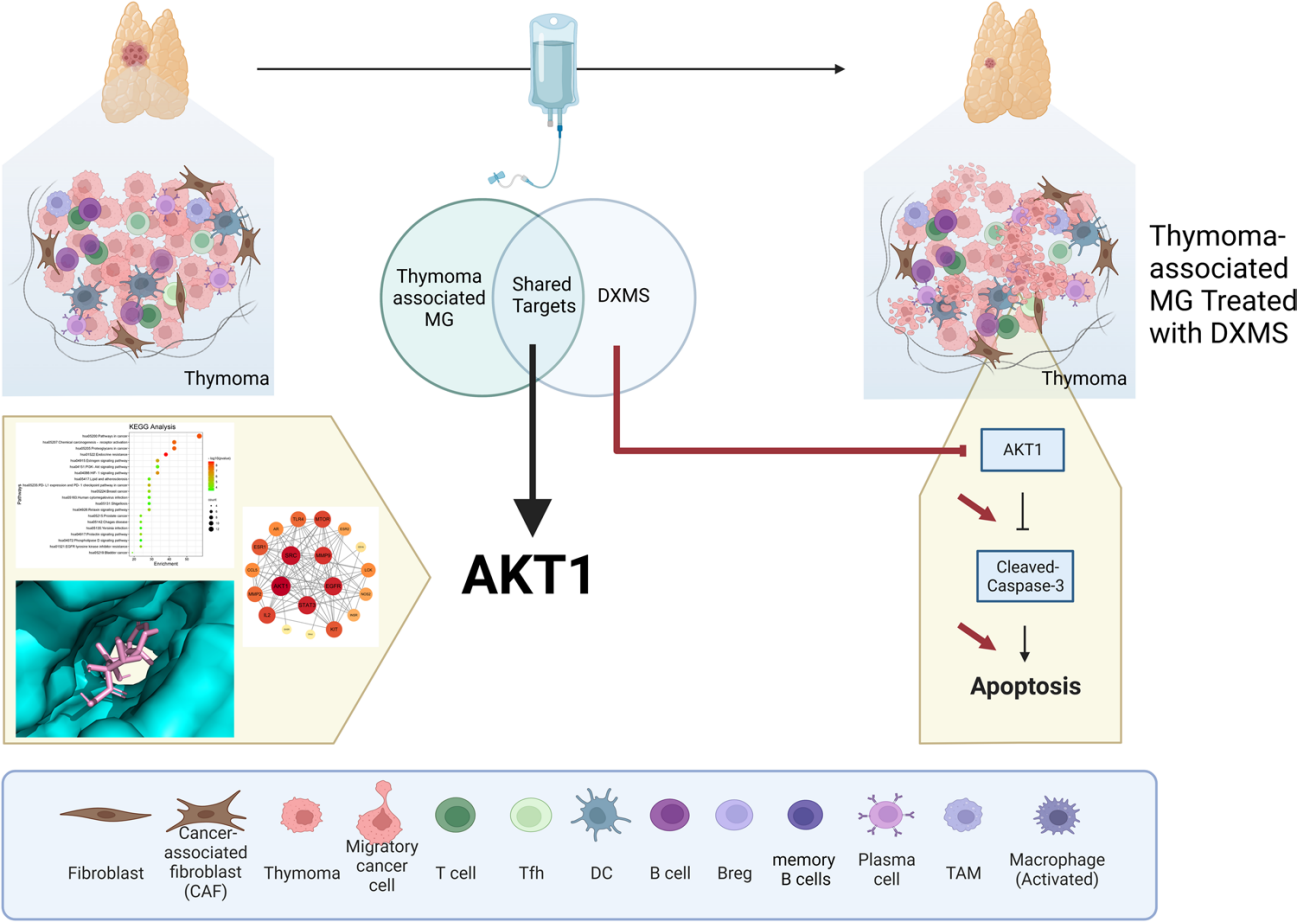
Overview of the interactions between DXMS and thymoma-associated MG. DXMS promoted the apoptosis of thymoma cell and improved thymoma-associated MG by regulating AKT signaling pathway

Our research screened out the core target genes AKT1, MMP9, STAT3, SRC, and EGFR of thymoma-associated M, and carried out molecular docking verification. We have also successfully verified that dexamethasone may act on thymoma-associated MG through the AKT1-mTOR pathway in clinical patients, which was rarely studied before. Our research provided a new therapeutic target for the treatment of thymoma-associated MG. We also hope that by understanding the mechanism of occurrence, we could better clarify the development process of this disease and further provide new perspective for drug treatment of this disease (Fig. 8).

### Supplementary Information

Below is the link to the electronic supplementary material.

Supplementary file1 (XLSX 14 KB)

## Data Availability

All data can be obtained from PharmMapper, Super-PRED, Human Gene Database, STRING, and DAVID.
